# The visual ecology of selective predation: Are unhealthy hosts less stealthy hosts?

**DOI:** 10.1002/ece3.8464

**Published:** 2021-12-20

**Authors:** Nina Wale, Rebecca C. Fuller, Sönke Johnsen, McKenna L. Turrill, Meghan. A. Duffy

**Affiliations:** ^1^ Program in Ecology, Evolution and Behavior Departments of Microbiology & Molecular Genetics and Integrative Biology Michigan State University Michigan USA

**Keywords:** bluegill, *Daphnia*, parasitism, *Pasteuria*, selective predation, visual ecology

## Abstract

Predators can strongly influence disease transmission and evolution, particularly when they prey selectively on infected hosts. Although selective predation has been observed in numerous systems, why predators select infected prey remains poorly understood. Here, we use a mathematical model of predator vision to test a long‐standing hypothesis about the mechanistic basis of selective predation in a *Daphnia*–microparasite system, which serves as a model for the ecology and evolution of infectious diseases. Bluegill sunfish feed selectively on *Daphnia* infected by a variety of parasites, particularly in water uncolored by dissolved organic carbon. The leading hypothesis for selective predation in this system is that infection‐induced changes in the transparency of *Daphnia* render them more visible to bluegill. Rigorously evaluating this hypothesis requires that we quantify the effect of infection on the visibility of prey from the predator's perspective, rather than our own. Using a model of the bluegill visual system, we show that three common parasites, *Metschnikowia bicuspidata*, *Pasteuria ramosa*, and *Spirobacillus cienkowskii*, decrease the transparency of *Daphnia*, rendering infected *Daphnia* darker against a background of bright downwelling light. As a result of this increased brightness contrast, bluegill can see infected *Daphnia* at greater distances than uninfected *Daphnia*—between 19% and 33% further, depending on the parasite. *Pasteuria* and *Spirobacillus* also increase the chromatic contrast of *Daphnia*. These findings lend support to the hypothesis that selective predation by fish on infected *Daphnia* could result from the effects of infection on *Daphnia*'s visibility. However, contrary to expectations, the visibility of *Daphnia* was not strongly impacted by water color in our model. Our work demonstrates that models of animal visual systems can be useful in understanding ecological interactions that impact disease transmission.

## Graphical abstract

The authors use a model of predator vision to assess why parasitized zooplankton more often fall prey to bluegill predators that uninfected prey.

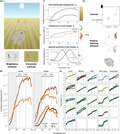

## 1 INTRODUCTION

When predators preferentially consume sick prey over healthy prey, a phenomenon called “selective predation,” they can substantially alter parasite transmission and evolution (Choo et al., 2003; Holt & Roy, 2007; Kisdi et al., 2013; Morozov & Adamson, 2011; Packer et al., 2003; Williams & Day, 2001). For example, when parasites need to be consumed to be transmitted (i.e., they are trophically transmitted), selective predation can promote parasite transmission; in contrast, predators that remove infectious hosts from the host population can depress transmission (Choo et al., 2003; Packer et al., 2003). Given that selective predation can have substantial impacts on both parasite and host fitness, we expect there to be a strong selection of the traits of infected hosts that cause predators to preferentially consume them. However, in many systems, it is unclear what these traits are or by how much they increase the probability that a host will be consumed. As a result, our ability to predict when selective predation will occur, or forecast its effects on the ecological and evolutionary dynamics of infectious diseases, remains limited.

One reason predators might selectively prey on infected hosts is that infection‐induced changes in the appearance of prey (which we refer to as visible symptoms) make them easier to detect. Parasites often induce changes in their hosts’ appearance—altering their body condition (Sánchez et al., 2018; Hall et al., 2007), shape (Roy, 1993), and color (Jones et al., 2016; Thünken et al., 2019; Wale et al., 2019; Williams & Cory, 1994; Zhou et al., 2016)—and it has been hypothesized that trophically, transmitted parasites manipulate their hosts so as to increase their chances of consumption (Thünken et al., 2019).

**FIGURE 1:**
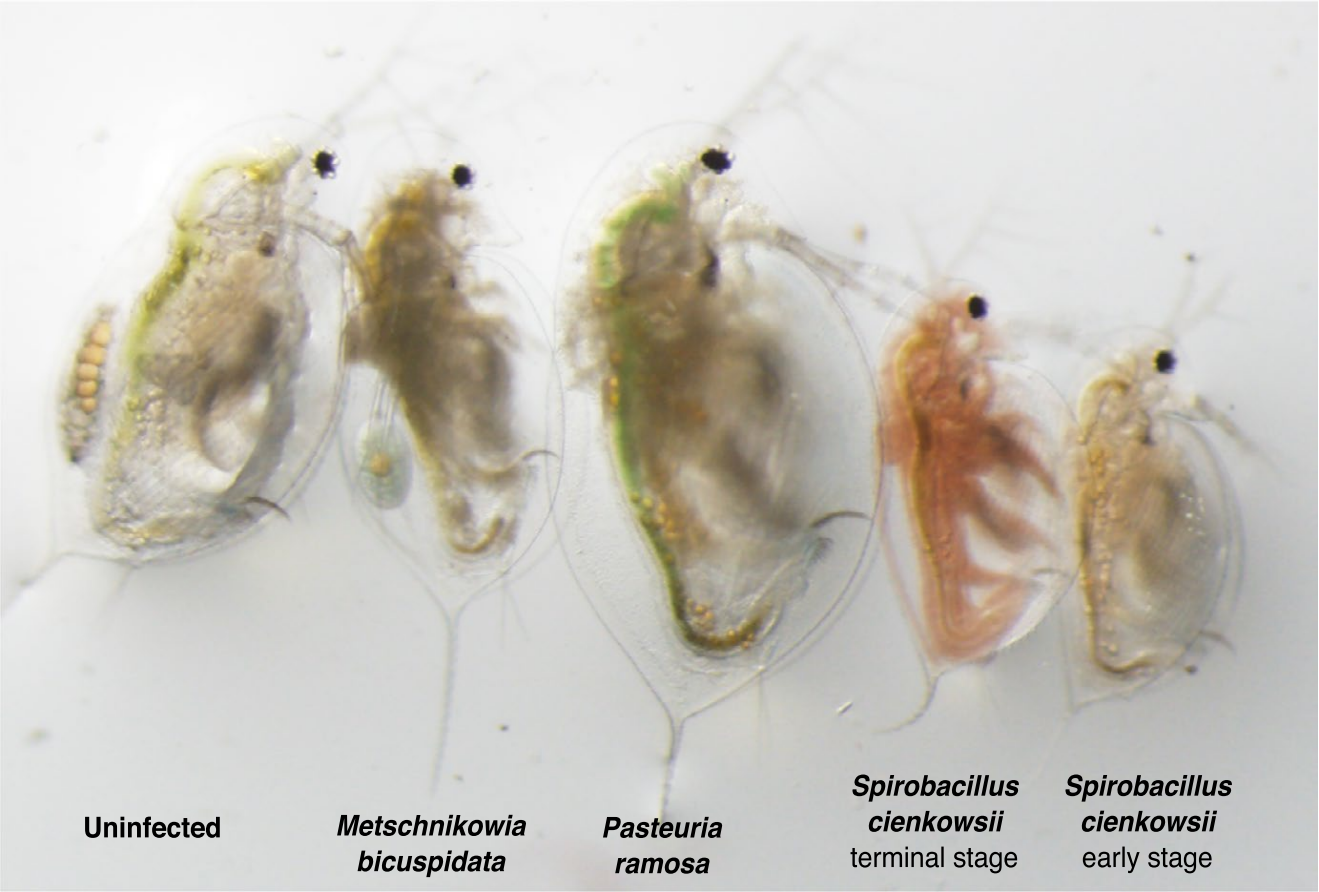
Parasites of *Daphnia* dramatically change their host's appearance. Infection with a variety of parasites (as labeled) induce distinctive symptoms in *Daphnia dentifera* and increase the likelihood of selective predation by bluegill sunfish (Duffy & Hall, 2008; Duffy et al., 2005). The symptoms of *Spirobacillus* infection change dramatically with infection stage

Quantitatively examining whether a particular visual symptom mediates selective predation is difficult for two reasons. First, parasites induce a variety of phenotypic changes in their hosts and many of these changes could increase their host's vulnerability to predation. For example, the bacterial parasite, *Spirobacillus cienkowskii*, simultaneously changes the transparency, color, and motility of its zooplankton host (Figure 1, Wale et al., 2019, Wale & Duffy *personal observation*). Therefore, it is not possible to disentangle the effect of one symptom of infection on predator selectivity from the effect of another, at least without experimentally manipulating prey symptoms. Second, differences in the visual systems of humans and other animals mean that we cannot reliably assess whether a visible symptom alters a predator's ability to detect prey. The human visual system differs from that of many other animals in the number and spectral sensitivity of photoreceptors. For example, humans have three photoreceptors, whereas birds have four, one of which is sensitive to ultraviolet (UV) light; as a result, birds “see” in the UV and may perceive objects very differently than humans (Olsson et al., 2018). The field of visual ecology has revealed that, because of these mismatches between human and animal visual systems, humans can overestimate the importance of visual signals that mediate ecological interactions—or, conversely, completely overlook them (Eaton, 2005; Matz et al., 2006). For this reason, we must take a “predator's eye view” as we seek to understand whether, and by how much, a particular visible symptom of infection alters interactions between predators and prey.

Here, we use a model of predator vision to quantitatively investigate whether one visual symptom of infection—a change in host transparency—could mediate selective predation in a fish–zooplankton–parasite system where predation is widespread, is selective, and has important epidemiological effects (Duffy & Hall, 2008; Duffy et al., 2005). *Daphnia* are partially transparent prey of visually hunting fish, such as bluegill sunfish (*Lepomis macrochirus*); their transparency is thought to have evolved as a means by which to avoid predators (Lampert, 2011). *Daphnia* are also host to a wide variety of parasites (Duffy et al., 2005; Ebert, 2005). Bluegill sunfish selectively prey on *Daphnia* infected by several parasites (Duffy et al., 2005, 2019; Duffy & Hall, 2008; Johnson et al., 2006), though their selectivity for infected hosts is abrogated in water with high concentrations of dissolved organic carbon (DOC) (Johnson et al., 2006). It has long been suspected that bluegill selectively prey on infected *Daphnia* because parasites fill up the hemolymph of *Daphnia*, blocking the penetration of light through them—that is, because parasites reduce *Daphnia*'s transparency. This hypothesis has not been rigorously tested, however. We used a model of the bluegill visual system to quantify the effect of three different parasites on *Daphnia*'s transparency and contrast, in water containing different concentrations of DOC. We found that infection dramatically reduces the transparency of *Daphnia* as perceived by a bluegill. As a result, the brightness contrast of *Daphnia* with their background increases and, with it, the distance at which bluegill can see infected *Daphnia* as compared to healthy hosts. Furthermore, two parasites (*Spirobacillus cienkowskii* and *Pasteuria ramosa*) also change the chromatic (i.e., color) contrast of *Daphnia* with their background. The extent to which infection changes the brightness and chromatic contrast of *Daphnia* varies with the parasite—*Spirobacillus cienkowskii* induces the greatest changes—but, surprisingly, not with water color. Our work thus lends strong support to the hypothesis that selective predation by bluegill is driven at least in part by the reduction in transparency of infected *Daphnia*.

## 2 METHODS

### 2.1 Approach

The extent to which an object contrasts with its background determines whether it is detectable to a viewer (e.g., dark blue ink is easier to see on white paper than on black). Therefore, quantifying the contrast of an object (or target, as we shall refer to it hereafter) is the central goal of any analysis aimed at understanding how detectable that target is.

**FIGURE 2:**
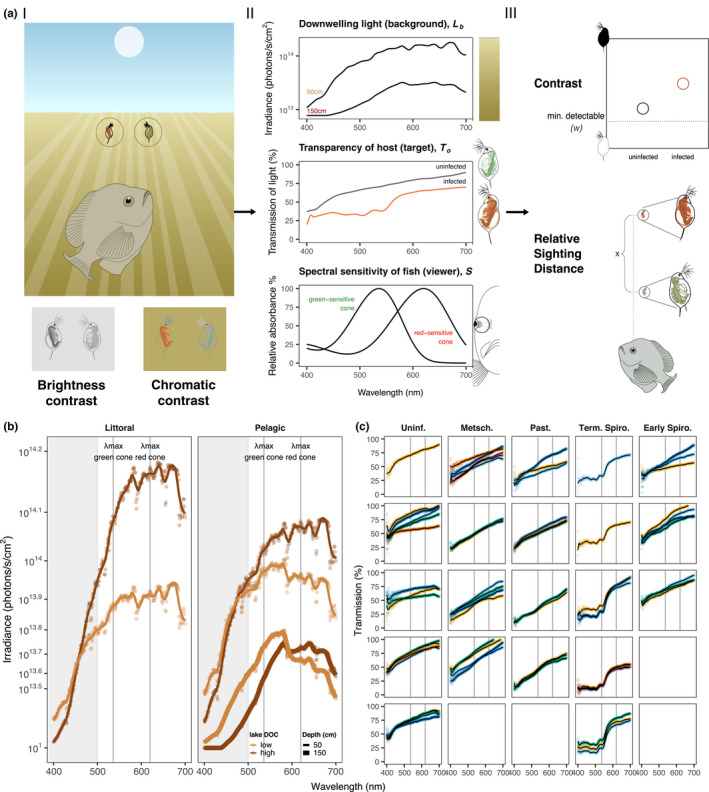
A visual ecology approach to understanding the impact of visual symptoms on predation. (a) I. To characterize how readily bluegill predators see *Daphnia* when they are looking up at them, we quantified the brightness and chromatic contrast of *Daphnia* with the downwelling light. II. To achieve this, we used spectroradiometry to measure the spectra of downwelling light in different lake environments (top panel—data displayed are those from the pelagic region of the high‐DOC lake; see b), and the transmittance of light through *Daphnia* tissues (middle—spectra displayed are of an uninfected and a terminal‐stage *Spirobacillus*‐infected host; see c). Data from Hawryshyn et al. (1988) were used to calculate the spectral sensitivity of the bluegill's two cones (bottom). III. To quantify the brightness and chromatic contrast of *Daphnia*, we integrated these data into a model of bluegill vision (Section 2.3) that accounts for the bluegill's capacity to detect the contrast between two stimuli, as determined by the contrast threshold (ω) of their photoreceptors. From this model, we also calculated how much further an infected vs. uninfected animal is detectable to a bluegill (the relative sighting distance). (b) The irradiance of downwelling light in the environment. The irradiance of downwelling light in the littoral (left) and pelagic (right) regions of two lakes that differ in DOC concentration. Raw data are given by points; smoothed data, as used in the analysis, by the line. Grey shaded area indicates the part of the spectrum most absorbed by DOC. (c) The spectra of light transmitted by uninfected and infected *Daphnia*. *Daphnia* were infected with *Metschnikowia* (Metsch.), *Pasteuria* (Past.) or *Spirobacillus* (Term. Spiro, Early Spiro). *Spirobacillus*‐infected animals change dramatically in color as the infection progresses from the early to the terminal (Term.) stage (see Figure 1). Each panel contains data from a single individual; raw data from each technical replicate are plotted in different colors with the smoothed spectra indicated by the line. In (b & c), the vertical lines indicate the wavelength of light to which the green‐sensitive and red‐sensitive cones of the bluegill are most sensitive (i.e., their *λ*_max_)

There are two ways that a target can contrast with the background—by how bright it is (brightness or achromatic contrast) and by how different it is in color (chromatic contrast) (Figure 2a.I). The target's contrast is first determined by the inherent light properties of the target and its background: How much light and of what spectrum does the target reflect back to the viewer's eye and how different is this light from the background? The second determinant is a function of the viewer—does the viewer have a visual system capable of detecting the contrast between the target and the background? To quantify the contrast of a target with its background in the eyes of a specific viewer, we thus need to combine information about the light properties of the target, background, and viewer. Models of animal visual systems allow us to do this.

Here, we use the model of Johnsen and Widder (1998) to understand whether, from the point of view of a bluegill predator, the accumulation of parasites within *Daphnia* alters their transparency and hence their brightness contrast. *Daphnia* comprise between a quarter and a half of the diet of adult bluegill (Mittelbach, 1984), which primarily hunt during the day using vision (Keast & Welsh, 1968; Douglas & Hawryshyn, 1990). We choose to model a specific hunting scenario, whereby a bluegill approaches the *Daphnia* from below so that the *Daphnia* is observed against the background of light coming from above the surface (i.e., downwelling light). Bluegill also prey upon *Daphnia* that they are positioned in front of or below them (Spotte, 2007). However, modeling this scenario is fraught with assumptions and requires a considerable amount of data, beyond that which we could collect. We therefore restricted our analysis to the scenario whereby the bluegill is observing the *Daphnia* from below.

The model integrates data on (a) the downwelling light that serves as the background against which *Daphnia* are seen, in the eyes of a bluegill looking up (Figure 2a.II, top), the capacity of downwelling light to transmit through uninfected and infected *Daphnia* (Figure 2a.II, middle), the capability of fish to detect the light coming from the background and the *Daphnia* (Figure 2a.II, bottom), and the contrast threshold of the fish's visual system—the minimum difference in brightness between two objects that an organism can detect (ω)—which determines whether the fish can detect the contrast between the *Daphnia* and their background (Figure 2a.III). With this model, we can estimate whether, in a particular body of water, infected *Daphnia* are differentially detectable from uninfected *Daphnia* so that bluegill might selectively prey upon them.

### 2.2 Data

#### 2.2.1 The background: downwelling light

We quantified light conditions in two lakes, North and Gosling (Livingston County, Michigan USA), which harbor both bluegill and our focal parasites. The two lakes differ in their content of dissolved organic carbon (DOC), which strongly absorbs UV, short‐ (“blue”), and mid‐ (“green”) wavelength light (as indicated by the grey shaded areas in Figure 2c) and hence shifts the appearance of lakes toward a yellow or brown color (Wetzel, 2001). Relative to a set of 15 study lakes in the region around the University of Michigan, Gosling Lake and North Lake contain relatively high (~13 mg/L) and low (~5 mg/L) concentrations of DOC, respectively (Rogalski and Duffy (2020), M.A. Duffy unpublished data); we hereafter refer to them as the “high‐DOC” and “low‐DOC” lakes. We quantified light conditions in two locations (pelagic and littoral) in each of these lakes.

In August 2018, we measured downwelling irradiance using a spectroradiometer (Ocean Optics S2000) connected to a patch cord (Ocean Optics QP400‐2 UV‐VIS), which was in turn connected to a cosine corrector (Ocean Optics CC‐3 DA). Bluegill feed nearly continuously during the day in the epilimnion of the water column (Keast & Welsh, 1968; Werner & Hall, 1988) and can often be seen feeding in the shallows of these lakes. We thus measured downwelling light in the upper part of the water column—at a depth of 50 cm in the littoral zone and at 50 and 150 cm in the pelagic zone. Due to the vertical migration of *Daphnia*, which rise around dusk and descend around dawn (Lampert, 2011), it is often thought that bluegill consume *Daphnia* only during dusk and/dawn periods, though Keast and Welsh (1968) found equivalent numbers of Cladocera in the stomachs of bluegill in the mid‐afternoon (3–5.30 p.m.) and early morning (5–9 a.m.), with peak stomach fullness occurring at 3 p.m. To minimize the variance between light measurements between lakes and depths caused by the changing in the direction and intensity of light as the sun was setting, we made our measurements between 3 and 6 p.m.

We acknowledge that measures of radiance, rather than irradiance, are normally used in models of visual systems. Our use of irradiance should not significantly impact our conclusions, however, because the shape of the spectra of downwelling irradiance and radiance (and so the relative sighting distance; see Equation 6) at shallow depths is very similar (Jerlov, 1976).

#### 2.2.2 The target: infected and uninfected *Daphnia*

We focused on three parasites that are common in Michigan lakes and that, at least to human eyes, reduce the transparency of *Daphnia*: the fungal parasite, *Metschnikowia bicuspidata*, and the bacterial parasites, *Spirobacillus cienkowskii* and *Pasteuria ramosa* (hereafter referred to by genus name only). In lakes, bluegill sunfish selectively prey upon *Metschnikowia*‐ and *Spirobacillus*‐infected hosts; in an environment with equal numbers of infected and uninfected *Daphnia*, the rate of predation on infected hosts is estimated to be nine (in the case of *Metschnikowia*) or three (in the case of *Spirobacillus*) times greater than the rate of predation on uninfected *Daphnia* (Duffy & Hall, 2008). To our knowledge, no one has recorded selective predation upon *Pasteuria*‐infected hosts.

To measure the inherent capacity of *Daphnia* to transmit light of different wavelengths, we measured light transmission through the thorax of uninfected *Daphnia dentifera* and *Daphnia dentifera* infected with our focal parasites (Table 1). Briefly, we connected a compound light microscope (Olympus BX53) to the aforementioned spectroradiometer via a patch cord and SMA connector, attached to the microscope's trinocular port. In turn, we then mounted each *Daphnia* onto a clear glass slide, removed the water around them so that they were immobilized, and placed them under the microscope's objective. We then illuminated the animal from below using the microscope's lamp and found the thorax, under 20× magnification. We made replicate readings of light transmission through each animal. These readings were calibrated by measurements of the penetration of light through water atop the slide.

**TABLE 1:** Details of hosts and parasites used in this study

	Host clone	Parasite strain	*n*
*Metschnikowia*	Standard	Standard	4
*Spirobacillus*, early‐stage	L6D9	Vasani	5
*Spirobacillus*, terminal‐stage	L6D9	Vasani	3
*Pasteuria*	Mid37	G18	4
Uninfected	L6D9	NA	5

Note

All hosts were of the species *Daphnia dentifera*. *Metschnikowia* and *Pasteuria* were cultured in the laboratory by collecting infected hosts, storing them in the fridge (*Metschnikowia*) or freezer (*Pasteuria*), then grinding them up and using them to infect new hosts, as described in Searle et al. (2016). *Spirobacillus* infections were similarly generated (Wale et al., 2019).

The infected *Daphnia* subjects we used were experimentally infected as part of long‐term efforts to maintain the three focal parasites in culture in the laboratory. Different clones of *Daphnia* are used to maintain these parasites (see Table 1). We used uninfected animals of the L6D9 clone, which are used to maintain *Spirobacillus* infections, as the uninfected subjects in this experiment. Therefore, our data do not account for any baseline between‐clone differences in the appearance of the *Daphnia* in the different infection treatments that could be perceived by a bluegill; no differences are perceptible to human eyes.

#### 2.2.3 The viewer: the bluegill visual system

Bluegill sunfish are dichromats with color vision (Hawryshyn et al., 1988; Hurst, 1953). They have two photoreceptors: a single cone that maximally absorbs light at a wavelength of 536 nanometers (“green‐sensitive” or mid‐wavelength‐sensitive [MWS] cone) and a double cone that maximally absorbs light at a wavelength of 620 nanometers (“red‐sensitive” or long‐wavelength‐sensitive [LWS] cone) (Hawryshyn et al., 1988; Northmore et al., 2007) (Figure 2a.II). With this visual system, bluegill can perceive both the brightness and hue of targets.

A key parameter of the visual system models used herein is the contrast threshold of each photoreceptor, which determines the minimum difference in brightness between two objects that an organism can detect using said photoreceptor. This parameter is inversely proportional to the signal‐to‐noise ratio of the photoreceptor used to see the object in question (Vorobyev et al., 2001; Vorobyev & Osorio, 1998).

The contrast threshold (ω) of the bluegill visual system has been estimated by two different authors, using two methodologies that differ in sensitivity. Northmore et al. (2007) used sine‐wave gratings to establish that the brightness contrast threshold of the entire bluegill visual system was 0.03, which means the minimum difference in the brightness of two objects that a bluegill can detect is 3%. Hawryshyn et al. (1988) used a more sensitive heart rate conditioning method to estimate the contrast threshold of each of the bluegill's two cones. This analysis yielded estimates of 0.003 for the mid‐wavelength‐sensitive (MWS or green‐sensitive) cone and 0.007 for the long‐wavelength sensitive (LWS or red‐sensitive) cone. Since the estimates of Hawryshyn et al. (1988) are an order of magnitude lower than estimates of brightness contrast thresholds in other freshwater fish (Douglas & Hawryshyn, 1990), we used Northmore et al. (2007)’s value (0.03) to calculate brightness contrast and relative sighting distance; we refer to this value as ω*_b_* (Equations 4, 5, and 6). Since the analysis of chromatic contrast required estimates of the contrast threshold of each photoreceptor (see Equation 7), we used Hawryshyn et al. (1988) estimates to calculate chromatic contrast (0.003, 0.007). We refer to these values as *ω_mws_* and *ω_mws_*, respectively.

### 2.3 Model

We adapt the model of Johnsen and Widder (1998) to investigate how infection alters the detectability of an infected vs. an uninfected *Daphnia* by a bluegill sunfish.

#### 2.3.1 Brightness contrast

The detectability of an object underwater is primarily determined by the extent to which it is brighter or darker than its background, that is, its brightness contrast (Johnsen, 2014). The contrast of an object, *o*, against a large background, *b*, in the context of a particular visual system is defined by the Weber contrast:

(1) $$C_{o}=\frac{Q_{o,p}‐Q_{b,p}}{Q_{b,p}}=\frac{Q_{o,p}}{Q_{b,p}}‐1$$

where *Q* is the quantum catch of the visual stimulus (as specified by the first subscript) by a particular photoreceptor, *p* (Johnsen, 2014). The quantum catch is defined as

(2) $$Q\propto \int_{\min}^{\max}L\left(\lambda \right)S\left(\lambda \right)d\lambda$$

where *L* is the spectrum of the radiance of the stimulus, and *S* is the spectral sensitivity of the photoreceptor at wavelength *λ* (i.e., the degree to which it absorbs light of said wavelength).

*Daphnia* are partially transparent animals. We define transparency (*T*) as a value between 0 (completely opaque) and 1 (completely transparent). When perceived from below, the light hitting the front on an animal and being reflected back to the viewer is scant. Hence, in this context, the contrast of an animal is determined by the extent to which the light that is illuminating the animal from above (downwelling light) can penetrate through it. The contrast of a *Daphnia* being seen from below is thus calculated per Equation 1, where *Q_o_* is defined as

(3) $$Q_{o}\propto \int_{\min}^{\max}L\left(\lambda \right)S\left(\lambda \right)Td\lambda$$

As such, *C_o_* spans from 0, where the *Daphnia* completely matches the bright, downwelling light, and −1, where it appears as a completely opaque silhouette against it.

To implement this model, we estimated the spectral sensitivity *S* of the cones from their wavelengths of maximal absorption (Section 2.2.3) according to the model of Govardovskii et al. (2000), using the pavo package in R (Maia et al., 2019), and integrated over the wavelengths from 400nm to 700nm, which encompasses the spectral sensitivity of the bluegill visual system. The radiance of downwelling light was used as *L* and the transmission of light through the *Daphnia* as *T*. Since *T* was measured at very short distance from the *Daphnia* (Section 2.2.2), *C_o_* is best interpreted as the inherent contrast of the *Daphnia,* that is, the contrast at zero distance from bluegill's eye Johnsen (2014).

Whether a target is detectable to a viewer depends on the relative magnitude of the object's contrast *C_o_* and the contrast threshold of the viewer's photoreceptors *ω_b_* (Siddiqi et al., 2004; Vorobyev & Osorio, 1998). In order to understand whether *Daphnia* are distinguishable from their background and, if so, by how much, we thus calculate the brightness contrast (*ΔS_b_*),

(4) $$\Delta S_{b}=\frac{\left|C_{o}\right|}{\omega_{b}}$$

following (Olsson et al., 2018 and Siddiqi et al., 2004). Brightness contrast has units of just noticeable differences (JNDs); a target is detectable when its brightness contrast exceeds 1 JND.

#### 2.3.2 Relative sighting distance

To set the measurements of brightness contrast of infected vs. uninfected *Daphnia* in further biological context, we used the contrast estimates to calculate the relative sighting distance of infected, *i*, vs. uninfected, *u*, *Daphnia*.

The maximum distance at which an object can be perceived (*d_sight_*) underwater is a function of (i) the inherent contrast of the object (*C_o_*) with its background, (ii) the viewer's contrast threshold (*ω_b_*), and (iii) the clarity of the water, as defined by two parameters the beam attenuation coefficient, *c*, and the diffuse attenuation coefficient, *K*, per the equation

(5) $$d_{\mathrm{sight}}=\frac{\ln\left(\frac{\left|C_{o}\right|}{\omega_{b}}\right)}{c‐K}$$

From this equation, we can derive the *relative* distance that an infected (*i*) vs. an uninfected (*u*) *Daphnia* can be perceived by a bluegill as

(6) $$R_{\mathrm{sight}}=\frac{\frac{\ln\left(\frac{\left|C_{i}\right|}{\omega_{b}}\right)}{c‐K}}{\frac{\ln\left(\frac{\left|C_{u}\right|}{\omega_{b}}\right)}{c‐K}}=\frac{\ln\left(\frac{\left|C_{i}\right|}{\omega_{b}}\right)}{\ln\left(\frac{\left|C_{u}\right|}{\omega_{b}}\right)}$$

**FIGURE 3:**
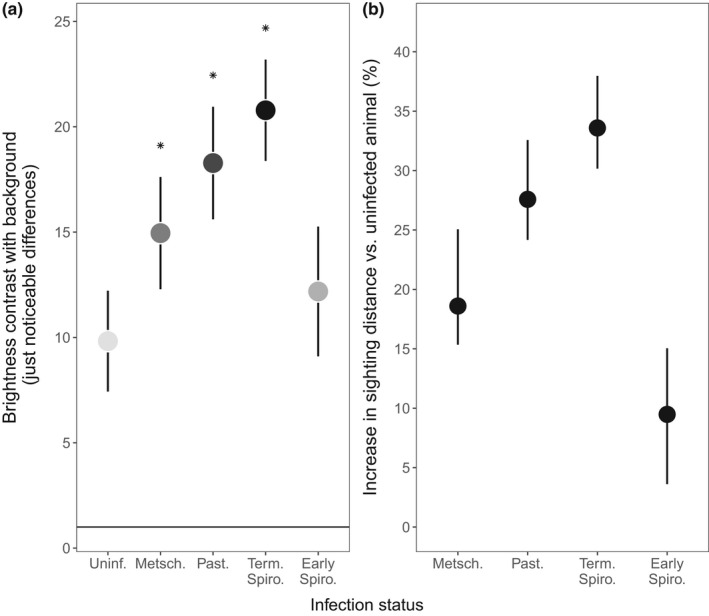
Parasites increase the detectability of *Daphnia* by reducing their transparency and so increasing their brightness contrast and sighting distance, relative to uninfected *Daphnia*. The (a) brightness contrast and (b) relative sighting distance of uninfected *Daphnia* and *Daphnia* infected with *Metschnikowia* (Metsch.), *Pasteuria* (Past.), and at the terminal and early stages of *Spirobacillus* infection (Term. Spiro. and Early Spiro., respectively). (a) Horizontal line indicates 1 JND: the smallest difference in brightness contrast that a bluegill can detect. Points and error bars represent means and 95% confidence intervals as estimated from the final statistical model of brightness contrast. Point fill indicates the appearance of *Daphnia* in the eyes of a bluegill (where white is transparent, black opaque), as estimated from a statistical model of *C_o_*. Stars indicate where the brightness contrast of *Daphnia* is significantly greater than that of uninfected *Daphnia*. (b) The relative sighting distance of infected *Daphnia* as compared to an uninfected *Daphnia*. Points and error bars represent means and 95% confidence intervals, as estimated by the resampling procedure described in the Methods section

For ease of interpretation, we translate the *Rsight* values we derive into the percent increase in the sighting distance in the text (Figure 3b).

To estimate the mean and 95% confidence interval around the estimates of relative sighting distance, we used a permutation approach. First, we calculated the raw contrast of each animal, at the level of the technical replicate. Second, we fit a statistical model, including the main effects of lake, depth, and treatment and a random effect of individual animal, to these data and extracted the fitted contrast values for each individual animal. Using these estimates, we calculated the numerator of the sighting distance equation for each individual. Relative sighting distance was then calculated by resampling the individuals without replacement in both the focal infected treatment and the uninfected treatment, finding the mean *dsight* numerator for each group and then their ratio. This procedure was repeated 1000 times to generate a distribution of *Rsight* values, and the 2.5% and 97.5% percentiles of this distribution were used as the confidence interval.

#### 2.3.3 Chromatic contrast

In addition to their brightness, *Daphnia* may contrast with their background in terms of their color. The distance in color space between a *Daphnia* and its background is given by the chromatic contrast (*ΔS_c_*)

(7) $$\Delta S_{c}=\sqrt{\frac{{\left(\Delta q_{\mathrm{lws}}‐\Delta q_{\mathrm{mws}}\right)}^{2}}{\omega_{\mathrm{lws}}^{2}+\omega_{\mathrm{mws}}^{2}}}$$

where *ω_lws_* and *ω_mws_* are the contrast thresholds of the two photoreceptors (cf. Section 2.2.3), and *Δq_p_* is the contrast perceived by each receptor *p*, and is given by

(8) $$\Delta q_{i}=\log\left(\left|Q_{o,p}\right|\right)‐\log\left(\left|Q_{b,p}\right|\right)$$

(Olsson et al., 2018; Vorobyev & Osorio, 1998), where *Q_o_*_,_ *_p_* and *Q_b_*_,_ *_p_* denote the quantum catches of the *Daphnia* and of the downwelling light, respectively, as in Equation 1.

As in the case of brightness contrast, chromatic contrast is measured in units of JNDs and the discriminability threshold is 1 JND. Whether two stimuli that are >1 JND different from their background are differentially conspicuous to the viewer remains a matter of debate (Fleishman et al., 2016; Santiago et al., 2020). Recent experiments suggest that the relative conspicuousness of two targets with suprathreshold chromatic contrasts (JND > 1) does increase with the difference in their JNDs (Fleishman et al., 2016; Santiago et al., 2020). However, Santiago et al. (2020) found that the ability of fish to discriminate between targets saturates as the targets’ contrast with the background increases, such that fish may not be able to discriminate between two objects that contrast greatly with their background, for example, by >20 JNDS. Since the contrast thresholds we use to calculate chromatic contrast are an order of magnitude smaller than those we use to calculate brightness contrast, our estimates of chromatic contrast are much greater than our estimates of brightness contrast. In light of the aforementioned debate, and because differences between these threshold estimates likely stem from the different methodologies used to estimate them (Douglas & Hawryshyn, 1990), we encourage the reader to be cautious in their interpretation of the absolute size of the chromatic contrasts but rather focus on the relative difference between treatments.

### 2.4 Statistical analysis

Statistical analysis was performed using R, version 4.0.4. We employed mixed‐effects models to analyze the brightness and chromatic contrast of *Daphnia* using the lmer and nlme packages, respectively.

#### 2.4.1 Random effects

To control for individual variation between *Daphnia*, experimental individual was included as a random effect.

Our dataset consists of both biological replicates (individual *Daphnia* within a treatment) and technical replicates (different measures of light transmission through a single *Daphnia*) (Figure 2c). Each of these technical replicates was then used to calculate the contrast of *Daphnia* in different environments. As a result, there is a correlation between the different contrast values calculated using each technical replicate (e.g., of the individual in each different environment), in addition to a correlation between values that pertain to each individual *Daphnia*. While the latter is accounted for using a random effect for individual in the model, the former is not and violates the assumption of the models used herein. An alternative analysis strategy that would account for the clustering of contrast calculations at the level of the technical replicate would be to employ a mixed‐effects model that specifies “technical replicate” as a random effect nested among “individual,” the environmental variables as crossed random effects, and infection treatment as the only fixed effect. We investigated whether such a model would yield different results in an analysis of brightness contrast but found that it did not: The same terms were significant, and their effect size was unchanged in any meaningful way. Because we are interested in the size of the effect of the environmental variables and their interaction with treatment—and because we had fewer than five technical replicates per *Daphnia*—we elected to present the results from the model including only the random effect of individual.

The fit of models was verified by visual inspection of residuals. In the analysis of chromatic contrast, we found that the residuals varied systematically with treatment. We thus used the nlme package to analyze chromatic contrast, since it permitted us to specify treatment as a variance covariant following Zuur et al. (2009).

#### 2.4.2 Fixed effects and model fitting

We built a full model that included the environmental parameters—depth, lake, and zone of the lake (pelagic vs. littoral)—and infection treatment as main effects. We included an infection treatment‐by‐lake interaction to investigate whether the effect of infection on the perceptibility of *Daphnia* changed with lake DOC (per Johnson et al. (2006)). Because depth greatly alters light environment (Figure 2b)—and hence potentially contrast—we also included a treatment‐by‐depth interaction.

To obtain a final model, which included only significant explanatory variables, we sequentially dropped insignificant terms using either Kenward–Roger's *F* test or likelihood‐ratio tests, for models of brightness and chromatic contrast, respectively. If a model term was insignificant but improved the AIC of the model, it was retained. To investigate whether the contrast of *Daphnia* harboring each parasite was different from uninfected animals, we performed post hoc comparisons using the emmeans package (Lenth et al., 2019). *p*‐values were corrected using the Dunnett adjustment for multiple comparisons.

### 2.5 Power analysis

We used the R package simr (Green & MacLeod, 2016) to investigate our ability to detect a 1 JND change in the brightness contrast of animals infected with different parasites as the lake environment changed (i.e., a treatment: lake interaction). We specified that the model be simulated 1000 times.

## 3 RESULTS

### 3.1 Brightness contrast

To a bluegill looking up at the water's surface, *Daphnia* appear as dark silhouettes against the background of bright downwelling light (Figure 3a; JND > 1). *Daphnia* contrast less with their background in the high‐DOC lake and in deeper water (brightness contrast *lake F*_1,379_ = 6.7, *p* = .01; brightness contrast *depth F*_1,379_ = 9, *p* < .01), but the effect of these environmental parameters is small (estimated reduction in contrast in the higher DOC lake = 0.5 JND, 0.12–0.85 95% CI; with depth = 0.6 JND, 0.2–0.99 95% CI) The contrast of *Daphnia* is unaffected by lake zone (i.e., pelagic vs. littoral; brightness contrast, *location F*_1,378_ = 1.5, *p* = .2).

Infected *Daphnia* are less transparent than uninfected *Daphnia* (Figure 2c). As a result, against a background of downwelling light, infected *Daphnia* appear darker than uninfected animals (Figure 3a, brightness contrast, *treatment F*_4,16_ = 14, *p* < .001) and bluegill are predicted to detect infected *Daphnia* at farther distances than healthy *Daphnia* (Figure 3b). How much further away a bluegill can detect an infected *Daphnia*, as compared to a healthy conspecific, is dependent on the infection's cause: The sighting distance of terminal‐stage *Spirobacillus*‐infected animals is 33% (on average, 95% CI = 30%–38%) greater than healthy animals, while the sighting distance of *Metschnikowia* animals is 19% (on average, 95% CI = 15%–25%) higher than healthy conspecifics. The great disadvantage of *Spirobacillus* infection, in terms of perceptibility to predators, only appears at the terminal stage of infection, however. *Daphnia* at the early‐stage *Spirobacillus* infection contrast with their background no more than healthy animals (post hoc analysis of brightness contrast, *p* = .5). Contrary to expectations, the effect of infection on brightness contrast (and hence sighting distance) is not different in lakes that vary in DOC (brightness contrast, treatment*lake F_4,370_ = 0.09, *p* = .98). However, a power analysis indicated that we had a limited ability to detect an impact of lake on the contrast of animals in different infection treatments (e.g., the probability of detecting a 1 JND change in the contrast of terminal‐stage *Spirobacillus*‐infected animals with lake was only 42%).

### 3.2 Chromatic contrast

**FIGURE 4:**
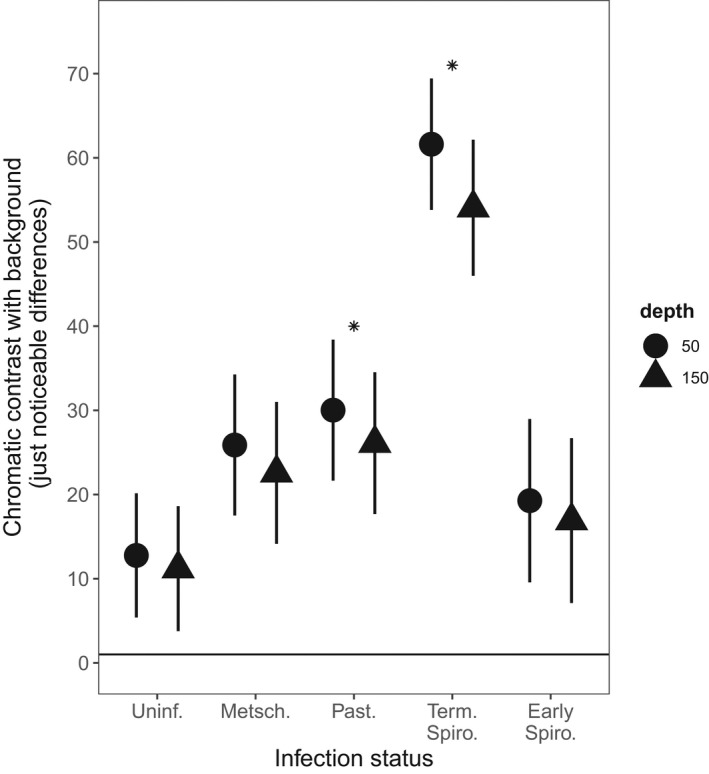
Terminal *Spirobacillus* and *Pasteuria* infections increase the chromatic contrast of *Daphnia*, particularly in shallow water. The horizontal line indicates the smallest difference in chromatic contrast that a bluegill can detect. Points and error bars represent means and 95% confidence intervals as estimated from the final statistical model. Stars indicate treatments in which the chromatic contrast of *Daphnia* is significantly greater than that of uninfected *Daphnia* in both lake environments. Depth is given in units of centimeters

Bluegill perceive *Daphnia* as a different color than the water in which they live (Figure 4; JND > 1). Infection further increases the chromatic contrast of *Daphnia* with their background, particularly in bright, shallow water (Figure 4; chromatic contrast, treatment*depth $\chi_{4}^{2}=14$, *p* = .01).

The effect of different parasites on the chromatic contrast of *Daphnia* is generally consistent with their effect on brightness contrast. The exception is that animals infected with *Metschnikowia* do not chromatically contrast with the background any more than uninfected hosts. The remaining findings are consistent with the brightness contrast findings. *Pasteuria*‐infected and terminal‐stage *Spirobacillus*‐infected *Daphnia* have a higher chromatic contrast than healthy animals (post hoc comparisons: *Pasteuria, p* = .03 and *Spirobacillus, p* < .001; Figure 4). Although *Spirobacillus*‐infected animals at the terminal stage of infection contrast greatly with the downwelling light (Figure 4), early‐stage *Spirobacillus*‐infected animals do not differ from healthy animals in terms of their chromatic contrast (post hoc comparison with uninfected animals, early‐stage *Spirobacillus*, *p* = .7; Figure 4). Finally, the effect of infection on the chromatic contrast of *Daphnia* does not change in different lake (i.e., DOC) environments (chromatic contrast, treatment*lake $\chi_{4}^{2}=2.8$, *p* = .6).

## 4 DISCUSSION

Selective predation by fish on infected *Daphnia* has been repeatedly demonstrated (Duffy & Hall, 2008; Duffy et al., 2005; Johnson et al., 2006) and can strongly influence epidemiological dynamics (Duffy & Hall, 2008; Duffy et al., 2005), but why predators select infected hosts had not been rigorously examined. Our analysis supports the hypothesis that parasites increase the visibility of *Daphnia* to bluegill predators by decreasing their transparency and, in the case of *Pasteuria* and terminal‐stage *Spirobacillus* infection, changing their color.

These changes in appearance are of such magnitude as to drive significant differences in the rate at which infected and uninfected animals are consumed (i.e., selective predation). We estimate that infection‐induced changes in brightness contrast increase the sighting distance of *Daphnia* by 19%–30%, relative to uninfected conspecifics, depending on the infection. Accordingly, given that the rate at which fish encounter *Daphnia* is proportional to the square of their sighting distance (Aksnes & Giske, 1993), fish could consume 40%–75% more infected *Daphnia* than uninfected *Daphnia* in a given period. It is more difficult to interpret the changes in chromatic contrast that occur with infection but, since bluegill preferentially feed on red objects over green objects, even when they are equally bright (Hurst, 1953), it is likely that this symptom also contributes to the selectivity of bluegill for infected animals.

While infections universally increase the visibility of *Daphnia*, some infections do so more than others. The different extent to which parasites change the brightness and chromatic contrast of *Daphnia* can be explained by their differential impact on the wavelengths of light to which bluegill are sensitive. Freshwater fish such as bluegill are thought to perceive brightness using the green‐sensitive cone. The tissues of infected *Daphnia* obstructed the penetration of light in the spectral region absorbed by this cone (Figure 2c), presumably because they were filled with parasites. Thus, the *Daphnia* are “silhouetted” against the bright downwelling light (Figure 3a). On the contrary, bluegill perceive the hue (and hence chromatic contrast) of objects by comparing the amount of light captured by the green‐sensitive and red‐sensitive cones. So it is the *difference* in the amount of light received by each cone that maximizes chromatic contrast. The spectrum of light transmitted by *Spirobacillus*‐infected hosts (and some *Pasteuria*‐infected hosts) changes rapidly in the spectral region that separates the peak absorbance of the bluegill's cones (as indicated by the gray vertical lines in Figure 2c). Thus, the chromatic contrast of *Spirobacillus*‐infected hosts is large as compared to uninfected and *Metschnikowia*‐infected hosts, which transmit light in a relatively constant manner in this region of the spectrum. Our finding that environmental variables have a negligible impact on the relative visibility of infected vs. uninfected *Daphnia* to bluegill can similarly be explained by looking at the features of the bluegill visual system. DOC absorbs UV, short‐ (“blue”) and mid‐ (“green”) light (300–500 nm) (Wetzel, 2001), but at the shallow depths we investigated, the effect of DOC is most apparent in the blue part of the spectrum (Figure 2b: shaded region). Neither of the bluegill's photoreceptors is particularly sensitive to light of this wavelength, so any change in the amount of light in this region will have had a limited impact on our estimates of *Daphnia*'s contrast and hence perceptibility.

Why then did Johnson et al. (2006) observe that the selectivity of bluegill sunfish for infected hosts changed with DOC, whereas our model predicts that it should not? The first explanation is that Johnson et al. (2006) used juvenile bluegill sunfish in their experiments, whereas our model focuses on the adult visual system. Unlike adults, juvenile *Lepomis* sp. have a visual system sensitive to changes in short‐wavelength and UV light, and hence to changes in DOC (Leech & Johnsen, 2003). Uninfected *Daphnia* scatter and reflect UV light and also absorb UV‐A light (Leech & Johnsen, 2006; White et al., 2005) and so are expected to contrast with UV light; how this contrast changes with infection is unknown. Nonetheless, if juvenile fish use a UV‐A sensitive cone to detect and select *Daphnia*, the concentration of DOC in water could change their foraging behavior and hence selectivity for infected *Daphnia*. That said, Leech and Johnsen (2006) found UV light had no effect on the foraging behavior of juvenile bluegill and theory suggests that temperate, freshwater fish should not use short‐wavelength light to forage because its intensity in their habitat changes markedly and frequently (Lythgoe, 1975). A second explanation for the discrepancy between our findings and those of Johnson et al. (2006) is that our model does not fully account for the impact of DOC on the sighting distance of *Daphnia*. The absolute sighting distance of an object is affected by several properties of the underwater light environment, including its spectrum and the rate at which it attenuates with distance, which determine the “color” and “amount” of light that reaches the viewer's eye, respectively (Johnsen, 2014). Since we were interested in the detectability of infected *Daphnia* relative to uninfected conspecifics and were unable to measure the attenuation of sunlight through lake water, we calculated the relative sighting distance of infected *Daphnia* (Equation 6). DOC changes both the color and attenuation rate of light underwater (and therefore the absolute and relative sighting distance of an object) (Wetzel, 2001). The effect of DOC on light attenuation could particularly impact bluegill feeding. For example, the rate at which bluegill feed on zooplankton decreases in the light‐limited environment induced by high DOC (Weidel et al., 2017) and even the much‐vaunted preference of bluegill for large size prey is abrogated in low light conditions induced by turbid water (Vinyard & O’brien, 1976). It may be that the absolute sighting distance is so limited in high‐DOC environments that relative changes in the sighting distance of infected vs. uninfected animals have little impact on feeding rates. Lastly, and relatedly, it is thought that in conditions of low light, bluegill may switch to hunting via lateral line sensing (Vinyard & O’brien, 1976). Clearly, a model of the bluegill visual system will not capture the effect of such a change in hunting behavior, whereas a behavioral experiment like that conducted by Johnson et al. (2006) might.

Indeed, our model has several assumptions that could limit its capacity to predict the behavior of bluegill in the wild. We used measurements of the transmission of light through the *Daphnia* thorax in our model. Thus, we implicitly assume that the entire *Daphnia* transmits light the same way that the thorax does, despite there being substantial spatial variation in the distribution of symptoms in infected hosts (Figure 1). Given that freshwater fish can select *Daphnia* according to the size of the eye (Branstrator & Holl, 2000; Zaret & Kerfoot, 1975) and the presence or absence of eggs (Johnson et al., 2006), it is not unreasonable to assume that the distribution of symptoms within a host might impact predator selectivity. Second, for technical reasons, we modeled a very specific hunting scenario, where the bluegill is looking up at the *Daphnia*, whereas bluegill also hunt while horizontally oriented with the prey in front of them (Spotte, 2007; Williamson & Keast, 1988). In this scenario, *Daphnia* would be observed against a background of sidewelling rather than downwelling light, which has a different spectrum and reduced intensity, and is subject to absorption and scattering by particulate matter on its way to the bluegill eye (Johnsen, 2014; Lythgoe, 1975). Though uninfected, transparent *Daphnia* contrast less with a background of sidewelling light (Loew & Lythgoe, 1978; White et al., 2005), it is difficult to intuit the impact of infection on *Daphnia's* perceptibility by bluegill in this scenario.

Our model, combined with the observations of Johnson et al. (2006), suggests that the visible symptoms of infection contribute to selective predation. This presents a quandary: These *Daphnia* parasites are obligate killers (Ebert (2005), Wale & Duffy *unpublished data*) that survive poorly in the bluegill gut (Duffy et al., 2005, 2019), so the fitness costs of inducing symptoms that increase the detectability of hosts could be substantial. Why then do these parasites induce such symptoms? Let us assume that phenotypes are in the control of the parasite (as we believe they are in the case of *Spirobacillus* (Bresciani et al., 2018)). The first hypothesis is it is merely a constraint of the system's biology—*Daphnia* are transparent, so occupying their hemolymph will naturally come at the cost of making them opaque. The second is that the production of symptoms puts parasites at risk of predation but that it is a risk worth taking. Were parasites to grow slower, and so reduce the symptoms that they induce, this could come at a disadvantage. In particular, slower growth might be costly in the context of within‐host competition with other parasite strains/species (de Roode et al., 2005) or surviving the *Daphnia* immune system (assuming a threshold model of immunity (Grossman & Paul, 1992)). Under this hypothesis, we would expect the frequency of “risky” symptoms to increase as the abundance of predators in the environment decreases. Intriguingly, in line with this expectation, *Pasteuria* strains induce a red color in their hosts in rock pools in Finland where fish predators are absent (D. Ebert, personal communication), and, conversely, in some lakes, terminal *Spirobacillus* infections tend to be white rather than red (Duffy & Wale *unpublished data*). Alternatively, selection might favor parasites that balance the benefits of symptoms with the risks by limiting the production of predation‐increasing symptoms to a small period of the infection, as in the case of *Spirobacillus*.

Here, we used a model of fish vision to precisely examine a long‐standing hypothesis about the mechanisms underlying selective predation in a fish–zooplankton–parasite system. We believe the tools and principles of visual ecology could advance our understanding of other parasite–host systems, too. Take, for example, trematode parasites that complete their life cycle by being trophically transmitted between multiple host species. Such parasites must reach a definitive host in order to reproduce and so incur a substantial cost if their intermediate host is consumed by a predator other than their definitive host (Mouritsen & Poulin, 2003). Visual ecologists have discovered that organisms can take advantage of the differences in the visual systems of different organisms to direct signals exclusively to a desired recipient (Cummings et al., 2003). This raises an intriguing question: Do trophically transmitted parasites exploit differences among predator visual systems to ensure that they reach the “right” host, for example, by inducing symptoms in their intermediate host that are visible to their definitive host, but not other predators? This example and the model herein demonstrate that integrating visual ecology and disease ecology could advance our understanding of the impact of symptoms on ecological interactions and disease transmission.

## CONFLICT OF INTEREST

The authors have no conflicts of interest to declare.

## AUTHOR CONTRIBUTIONS

**Nina Wale:** Conceptualization (lead); formal analysis (lead); investigation (lead); methodology (lead); visualization (lead); writing—original draft (lead); writing—review and editing (lead). **Rebecca C. Fuller:** Data curation (supporting); investigation (supporting); methodology (supporting); resources (supporting); software (supporting); writing—review and editing (equal). **Sonke Johnsen:** Resources (supporting); supervision (supporting); writing—review and editing (supporting). **McKenna L. Turrill:** Funding acquisition (supporting); investigation (supporting); methodology (supporting). **Meghan A. Duffy:** Conceptualization (supporting); funding acquisition (lead); investigation (supporting); methodology (supporting); supervision (lead); writing—review and editing (lead).

## Data Availability

The data can be accessed via datadryad.org at https://doi.org/10.5061/dryad.dv41ns20h.
